# 

**Published:** 1878-01-20

**Authors:** 


					﻿6
a
Ph
W
a
Ph
PP
o
I—I
A
a
S
a
a
o
o
H
a
<1
Ph
H"*}*
o
r-j
□D
I S
I a
o
CD
l-H
A
O
a
w
H
H
£
a
GO
a
a
a
a
co
<1
a
a
a |
voL.yiii.*/' January 20,1878. Noi.
THE SOUTHERN	O
MEDICAL RECORD.
ft ^AoNTHLY jJoUI^NAL OF PRACTICAL ^AeDICINE.
'■Terms; $2per annum in advance. Club lyates,§ copies $8 Single copies 20c.
“ QUICQUID PllSECIPIES ESTO BRET IS."
I
QD
A
O
W
H
O
o
PR
<*■4
•—♦
l-H
Q
H
l-H
o
>2*
CK>
r*
w
hs
o
»—i
O
-h
l-H
H
W
te*
CK
CO
o
r
l-H
o
l-H
H
ESTABLISHED IN 1870.
ATLANTA, GA.:
Sunny South Steam Publishing Uousi.
Address R. C. WORD, M. D., Managing Editor, Atlanta, Ga.
				

